# Comparative genomic analysis of *Leishmania (Viannia) peruviana* and *Leishmania (Viannia) braziliensis*

**DOI:** 10.1186/s12864-015-1928-z

**Published:** 2015-09-18

**Authors:** Hugo O. Valdivia, João L. Reis-Cunha, Gabriela F. Rodrigues-Luiz, Rodrigo P. Baptista, G. Christian Baldeviano, Robert V. Gerbasi, Deborah E. Dobson, Francine Pratlong, Patrick Bastien, Andrés G. Lescano, Stephen M. Beverley, Daniella C. Bartholomeu

**Affiliations:** Laboratório de Imunologia e Genômica de Parasitos, Instituto de Ciências Biológicas, Universidade Federal de Minas Gerais, Belo Horizonte, Brazil; Department of Parasitology, U.S. Naval Medical Research Unit No. 6, Lima, Peru; Universidad Peruana Cayetano Heredia, School of Public Health and Management, Lima, Peru; Department of Molecular Microbiology, Washington University School of Medicine, St. Louis, Missouri USA; Centre Hospitalier Universitaire de Montpellier, Departement de Parasitologie-Mycologie, Centre National de Reference des Leishmanioses, Montpellier, France

## Abstract

**Background:**

The *Leishmania (Viannia) braziliensis* complex is responsible for most cases of New World tegumentary leishmaniasis. This complex includes two closely related species but with different geographic distribution and disease phenotypes, *L. (V.) peruviana* and *L. (V.) braziliensis*. However, the genetic basis of these differences is not well understood and the status of *L. (V.) peruviana* as distinct species has been questioned by some.

Here we sequenced the genomes of two *L. (V.) peruviana* isolates (LEM1537 and PAB-4377) using Illumina high throughput sequencing and performed comparative analyses against the *L. (V.) braziliensis* M2904 reference genome. Comparisons were focused on the detection of Single Nucleotide Polymorphisms (SNPs), insertions and deletions (INDELs), aneuploidy and gene copy number variations.

**Results:**

We found 94,070 variants shared by both *L. (V.) peruviana* isolates (144,079 in PAB-4377 and 136,946 in LEM1537) against the *L. (V.) braziliensis* M2904 reference genome while only 26,853 variants separated both *L. (V.) peruviana* genomes.

Analysis in coding sequences detected 26,750 SNPs and 1,513 indels shared by both *L. (V.) peruviana* isolates against *L. (V.) braziliensis* M2904 and revealed two *L. (V.) braziliensis* pseudogenes that are likely to have coding potential in *L. (V.) peruviana*. Chromosomal read density and allele frequency profiling showed a heterogeneous pattern of aneuploidy with an overall disomic tendency in both *L. (V.) peruviana* isolates, in contrast with a trisomic pattern in the *L. (V.) braziliensis* M2904 reference.

Read depth analysis allowed us to detect more than 368 gene expansions and 14 expanded gene arrays in *L. (V.) peruviana*, and the likely absence of expanded amastin gene arrays.

**Conclusions:**

The greater numbers of interspecific SNP/indel differences between *L. (V.) peruviana* and *L. (V.) braziliensis* and the presence of different gene and chromosome copy number variations support the classification of both organisms as closely related but distinct species.

The extensive nucleotide polymorphisms and differences in gene and chromosome copy numbers in *L. (V.) peruviana* suggests the possibility that these may contribute to some of the unique features of its biology, including a lower pathology and lack of mucosal development.

**Electronic supplementary material:**

The online version of this article (doi:10.1186/s12864-015-1928-z) contains supplementary material, which is available to authorized users.

## Background

Leishmaniasis is a neglected tropical disease caused by a group of digenetic protozoan belonging to the genus *Leishmania*. It is transmitted by the bite of an infected female phlebotomine sand fly belonging to the genus *Lutzomyia* in the New World and *Phlebotomus* in the Old World [1]. Leishmaniasis is endemic in 98 countries and causes more than 1.5 million cases per year with more than 350 million people at risk [2, 3].

Leishmaniasis presents a wide spectrum of clinical manifestations that ranges from cutaneous leishmaniasis (CL) that affects tissues near the sand fly bite to mucosal leishmaniasis (ML) that is characterized by a progressive ulceration at the nares and nasal septum to the lethal visceral leishmaniasis (VL) that disseminates to visceral organs causing hepatomegaly, splenomegaly and even death [3, 4].

The *L. (V.) braziliensis* complex is one of the most important *Leishmania* group in the New World [5]. It comprises two closely related species (*L. (V.) peruviana* and *L. (V.) braziliensis*) [6], although there is some controversy regarding their status as distinct species [6]. As currently defined, *L.(V.) peruviana* is an endemic species in Peru with a limited distribution range within the Andean and inter-Andean valleys with some narrow areas of sympatry with *L. (V.) braziliensis* [7, 8].

*L. (V.) peruviana* causes CL and has been isolated from peridomestic mammals including dogs, mice and opossums, revealing its zoonotic status [9]. *L. (V.) braziliensis* is widely distributed in South America, although primarily in the Amazon Basin, and is referred as an anthropozoonosis [10]. *L. (V.) braziliensis* infections have a substantially higher potential to manifest as ML than any other new world leishmaniasis species, including *L. (V.) peruviana* [3, 11]. However, the parasite genetic factors that contribute to the differences in the pathogenesis of these two species are not well known.

Next generation sequencing has provided several advantages for characterizing species-specific traits across the genomes of several organisms. In *Leishmania* it has allowed to rapidly and comprehensively analyze a wide range of mutation types, including gene copy number variations (CNV) and aneuploidy [12]. Recently, CNV and expansions in tandem gene arrays have been proposed as a mechanism to increase gene expression with numerous species-specific gene amplifications [12]. These studies have suggested that extensive variation among duplicated tandem gene arrays plays a role in higher expression of their products and a diversification process in amplified genes [13]. Moreover, analysis of the chromosomal content from different cells within the same isolate have led to conclude that *Leishmania* presents a mosaic structure that may contribute to gene expression changes in response to environmental alteration modulating parasite phenotypes [12, 14].

In this study, we have conducted a comparative genomics analysis of two L*. (V.) peruviana* isolates against the reference genome M2904 of *L. (V.) braziliensis*. Comparative assessments have shown important differences in chromosome and gene copy number between both species. These analyses may serve to improve our understanding of parasite variation between these two closely related species that could be linked to their different disease phenotypes and to provide further insights into their status as distinct species.

## Results and Discussion

### Genome assembly

We used a combined *de novo* and reference based assembly approach (Baptista et al. in preparation) to generate a draft genome for each strain. *L. (V.) peruviana* mapped reads showed an overall 92.51 % mapping rate for PAB-4377 and 95.87 % for LEM1537 against *L. (V.) braziliensis*. Median genome coverage estimated from mapped reads into 6,899 single copy genes was of 59.1 and 35.0 for PAB-4377 and LEM1537, respectively.

The *L. (V.) peruviana* assemblies resulted in 28.51 and 25.27 megabases that were generated from 11,504 and 29,816 contigs in PAB-4377 and LEM1537, respectively. The resulting ordered assemblies consisted of 37 pseudo-chromosomes, due to the split of chromosome 20 in the *L. (V.) braziliensis* M2904 reference genome (LbrM.20.1 and LbrM.20.2) and a pseudo-chromosome containing unordered scaffolds (Chromosome 0).

The overall identity between *L. (V.) braziliensis* and *L. (V.) peruviana* calculated with MUMmer [15] confirmed the close relationship between *L. (V.) braziliensis* and *L. (V.) peruviana* (identity of 87.58 % for PAB-4377 and 77.1 % for LEM1537), and a closer relationship between the two *L. (V.) peruviana* isolates (99 %).

### SNP and Indel comparisons

Variants were identified following filtering for quality, read depth and haplotype score as described in the methods.

Comparisons identified 144,079 and 136,946 variants between *L. (V.) braziliensis* and *L. (V.) peruviana* PAB-4377 (115,851 SNPs and 28,228 Indels) and *L. (V.) peruviana* LEM1537 (108,826 SNPs and 28,120 Indels), respectively. Of these; 94,070 variants were shared between the two *L. (V.) peruviana* isolates. In contrast, the two *L. (V.) peruviana* isolates showed fewer variants among them (26,853). This finding is consistent with the high similarity obtained with MUMmer3 between both *L. (V.) peruviana* isolates and the greater difference with *L. (V.) braziliensis*.

Our results show that there is significant genetic differentiation between *L. (V.) braziliensis* and *L. (V.) peruviana* while intra *L. (V.) peruviana* variation is substantially lower. For comparison, a previous comparative study between *L. (L.) infantum* and *L. (L.) donovani* reference genomes found that 156,274 nucleotide changes differentiate between these closely related species [16], comparable to what we describe here for *L. (V.) braziliensis* and *L. (V.) peruviana*.

We then focused on the 94,070 variants from *L. (V.) braziliensis* that were shared by the two *L. (V.) peruviana* lines. Of these; 26,750 SNPs were located in 6,114 coding DNA sequences (CDS) (Additional file 1: Table S1). Of these, 14,244 SNPs (53.24 %) were synonymous mutations and 12,462 (46.59 %) were non-synonymous mutations. Additionally, eight SNPs mutating the annotated start codon (0.03 %) and 36 mutating the annotated stop codon were found (0.13 %). Most genes with high counts of SNP are hypothetical proteins, kinases and trafficking proteins stressing the need to characterize the function of these variable proteins (Table 1).

**Table 1:** Top ten high SNP count genes in two *L. (V.) peruviana* isolates

Gene ID	Annotation	Number of SNP	Gene length	CN PAB LEM	
LbrM.33.3060	Hypothetical proteins	135	14,943	0.97	0.87
LbrM.30.2340		83	11,340	0.98	0.75
LbrM.34.5330		82	19,875	1.07	1.25
LbrM.16.0180		69	13,302	0.82	0.79
LbrM.35.1580		68	16,767	1.01	0.76
LbrM.14.0770		63	12,570	0.68	0.39
LbrM.35.3160		43	12,582	1.05	0.98
LbrM.30.2160	Endosomal trafficking protein RME-8, putative	40	7335	1.20	1.19
LbrM.02.0130	Phosphatidylinositol kinase related protein, putative	39	14,775	0.51	0.47
LbrM.30.1620	protein kinase, putative	38	5112	1.30	1.23

Top ten genes showing high SNP differences in *L. (V.) peruviana* compared with *L. (V.) braziliensis* orthologs. Number of SNP and gene length are presented in nucleotides. Copy number (CN) estimated for the haploid genome of PAB-4377 (PAB) and LEM-1537 (LEM)

Variant calls for indels shared by both isolates detected 1,513 sites distributed in 408 CDS (Additional file 1: Table S2). Of these, 1,014 (67.0 %) were codon deletions, 146 (9.6 %) were insertions, 351 (23.2 %) frameshifts and two stop codons (0.1 %) were gained. Genes with most bases affected by indels include hypothetical proteins, kinesins and a lysine transport protein (Table 2).

**Table 2:** Top ten high INDEL count genes in L. (V.) peruviana

Gene ID	Annotation	Affected nucleotides	Gene length	CN PAB LEM	
LbrM.17.0390	Hypothetical proteins	57	3480	0.63	0.52
LbrM.21.1080		42	2895	0.76	0.56
LbrM.15.1180	Nucleoside transporter 1, putative	28	1848	1.34	1.33
LbrM.34.2710	Hypothetical protein	24	2133	1.43	1.6
LbrM.14.0785	Kinesin, putative	21	957	0.75	1.02
LbrM.31.1470	Hypothetical proteins	21	4089	0.82	0.66
LbrM.32.3450		21	2469	0.74	0.54
LbrM.33.2950		21	3582	0.91	0.77
LbrM.07.1050	RNA binding protein-like protein	19	1377	1.02	1.21
LbrM.25.1000	Hypothetical proteins	18	19,518	0.56	0.33

Top ten high indel count genes in *L. (V.) peruviana* compared with *L. (V.) braziliensis* orthologs. Gene length is presented in nucleotides. Copy number (CN) estimated for the haploid genome of PAB-4377 (PAB) and LEM-1537 (LEM)

Analysis of potential diagnosis targets that could accurately differentiate *L. (V.) peruviana* from *L. (V.) braziliensis* resulted in 270 genes with high SNP density regions between both species (Additional file 2, Additional file 1: Table S3). While most of these genes are hypothetical proteins, they could serve to design better molecular diagnosis tools to discriminate between these closely related species.

Two *L. (V.) braziliensis* pseudogenes (LbrM.04.0060, LbrM.28.2130) appeared to be functional in *L. (V.) peruviana*. LbrM.28.2130 codes for an X-pro, dipeptidyl-peptidase, serine peptidase and has orthologs in other *Leishmania* species from the Old and New World suggesting that it could be functional in *L. (V.) peruviana*. Peptidases have an important role in parasite survival, invasion, metabolism and host-parasite interaction [17], highlighting the importance of confirming coding function of this potential gene. LbrM.04.0060 codes for a putative pteridine transporter and shares 84 % identity with a folate/biopterin in *L. infantum*. It has been shown that *Leishmania* are pteridine auxotrophs and rely on a network of folate and biopterin transporters. Pteridine levels have a strong influence on metacyclogenesis in *L. (L.) major* [18].

### Chromosome copy number variation

**Fig. 1:**
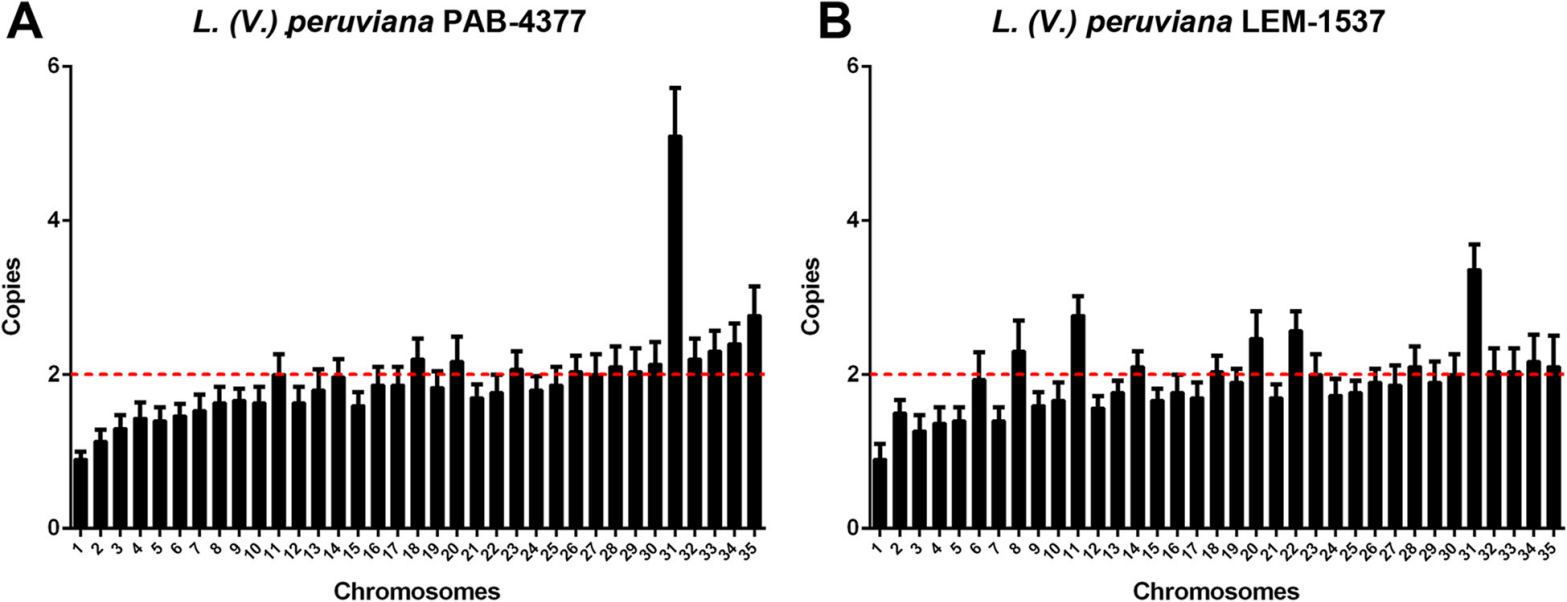
Chromosome copy number in *L. (V). peruviana.* Chromosome copy number variation in *L. (V.) peruviana*. **a** PAB-4377; **b** LEM-1537. Boxes represent the estimated copy number for each chromosome and standard deviation from the three methods. Mean genome ploidy is indicated by a dotted red line

Chromosome numbers were estimated by the average read density to each chromosome, and normalized to an assumed overall genome ploidy of 2n. Normalized chromosome copy number clustered around “disomy” although with significant departures from non-integral values evident for some chromosomes (Fig. 1). This finding is particularly important since the *L. (V.) braziliensis* M2904 strain is mostly trisomic [12].

**Fig. 2:**
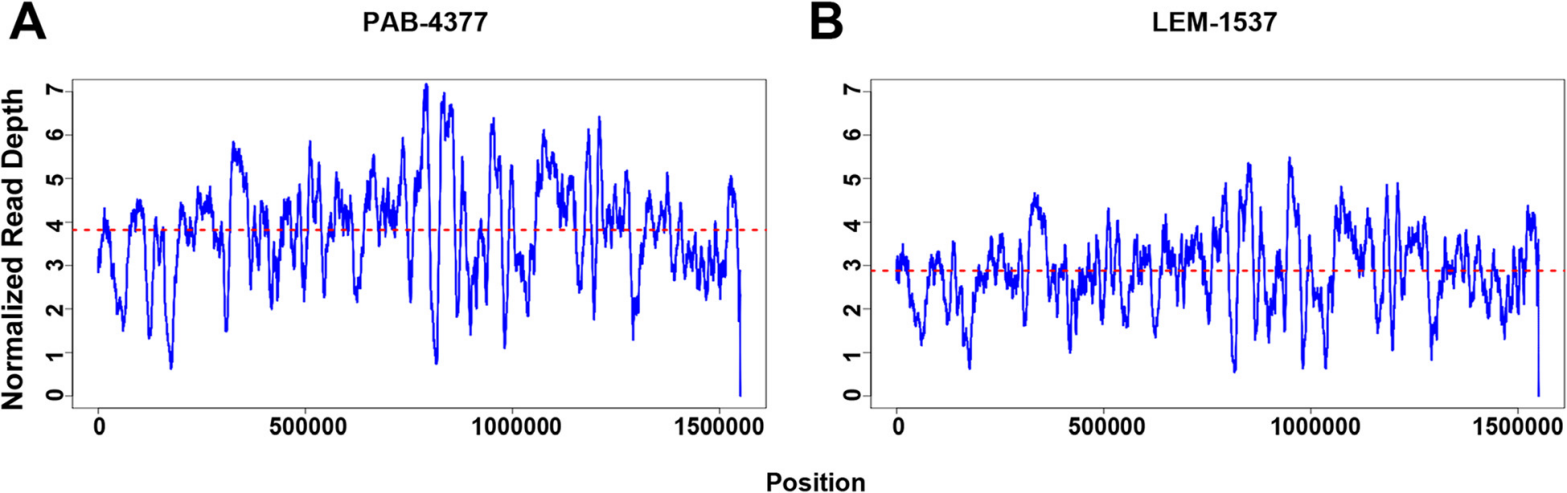
Chromosome 31 normalized read depth. Normalized read depth for supernumerary chromosome 31. **a** PAB-4377; **b** LEM-1537. Estimated ploidy indicated by a dotted red line. Blue lines represent normalized read depth for each position at the chromosome

The most pronounced departure from disomy occurred in chromosome 31, which presented a read depth between tetrasomy to hexasomy in PAB 4377 and trisomy in LEM1537 (Fig. 1, Additional files 3 and 4). In both isolates, read depth was evenly distributed along the entire sequence of Chr31, arguing against region-specific amplification (Fig. 2).

In both samples, chromosomes 1–5 and 7 appear to be closer to monosomy. This characteristic has also been estimated for chromosomes 1 and 3 of *L. (L.) mexicana* [12]. Interestingly, the pattern of aneuploidy involving chromosomes 8, 11, 20 and 22 in LEM1537 and 35 in PAB-4377 is different from the median ploidy of the rest of the chromosomes in both samples. These chromosomes appear to have intermediate read depth between disomic and trisomic profiles, suggesting a mosaic ploidy within the cell population (Fig. 1).

It has been suggested that mosaic aneuploidy could be a mechanism of rapid parasite adaptation in response to environmental changes within its host [14] and it has been shown to occur in closely related strains [16]. However, its origin in *Leishmania* remains to be investigated [16].

A second approach for assessing chromosome number is based upon allele frequencies. For disomic chromosomes, heterozygous SNPs should cluster around 50 %, while trisomic chromosomes should show two peaks at 33 and 67 % and tetrasomics at 25, 50 and 75 %, [12].

**Fig. 3:**
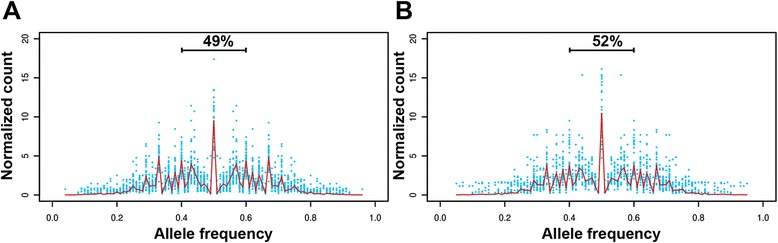
Normalized allele frequency distribution. Normalized allele frequency counts for *L. (V.) peruviana*. **a** PAB-4377; **b** LEM-1537. Blue dots show normalized counts at heterozygous positions for all disomic chromosomes. Mean count at each allele frequency is indicated by a red line. Cumulative percentage between 0.4 and 06 heterozygous frequencies support disomic tendency of most chromosomes

Allele frequency counts for each predicted heterozygous SNP further confirmed the overall disomic tendency (Fig. 3) and the highly heterogeneous structure within the cell populations with chromosomes presenting mixtures of allele profiles (Additional files 5 and 6).

Chromosomes with discordance between read depth analysis and their allele frequencies included chromosome 5, 7, 13, 17 and 19 that presented a tetrasomic or a mixture of trisomic and disomic patterns in PAB-4377 (Additional file 5).

In LEM1537, chromosomes 6 and 9 did not have a marked allele frequency pattern and chromosome 11, 14 and 25 presented discordance between read depth and allele frequencies (Additional file 6). Additionally, chromosomes 22, 23, 28 and 34 presented mixtures of disomy and monosomy that corresponded with their estimated read depth (Additional file 6, Fig. 1).

Discordance between allele frequency and read depth may be explained by cells presenting a high variation in their ploidies due to chromosome mosaism as has been previously suggested [12].

Interestingly, chromosome 31 that has been identified as supernumerary in both isolates appears to have disomic pattern (Additional files 5 and 6). This chromosome has been previously described as supernumerary in all *Leishmania* species [12]. It may be possible that this chromosome accumulates mutations in disomic alleles as has been reported in other chromosomes with the same pattern in *L. (L.) mexicana* [12].

Ontology analysis in the supernumerary chromosome 31 showed that this chromosome is enriched in genes involved in iron metabolism and other related molecular functions (Table 3, Additional file 1: Table S4). Iron sulfur proteins (Fe-S) are crucial for life since they mediate oxidation-reduction reactions during mitochondrial electron transport and are involved in the synthesis of amino acids, biotin and lipoic [19].

**Table 3:** Ontology analysis for chromosome 31

Go ID	Description	Corrected *p*-value
51,537	2 iron, 2 sulfur cluster binding	1.08E-03
9055	electron carrier activity	1.85E-02
4198	calcium-dependent cysteine-type endopeptidase activity	1.85E-02
51,536	iron-sulfur cluster binding	1.85E-02
51,540	metal cluster binding	1.85E-02
4148	dihydrolipoyl dehydrogenase activity	1.85E-02
4197	cysteine-type endopeptidase activity	3.81E-02
8234	cysteine-type peptidase activity	4.94E-02

Biosynthesis of Fe-S proteins is highly dependent on iron regulation in the cell [20]. Interestingly, ferrous iron transporters located in chromosome 31 have been described in *Leishmania* and they appear to be important for growth, replication and pathology, further stressing this connection [21, 22].

A sustained copy number increase in chromosome 31 among all *Leishmania* species [12] could serve as a mechanism to facilitate iron uptake and increase gene dosage of Fe-S proteins in an oxidative stressed environment.

### Gene copy number variation

**Fig. 4:**
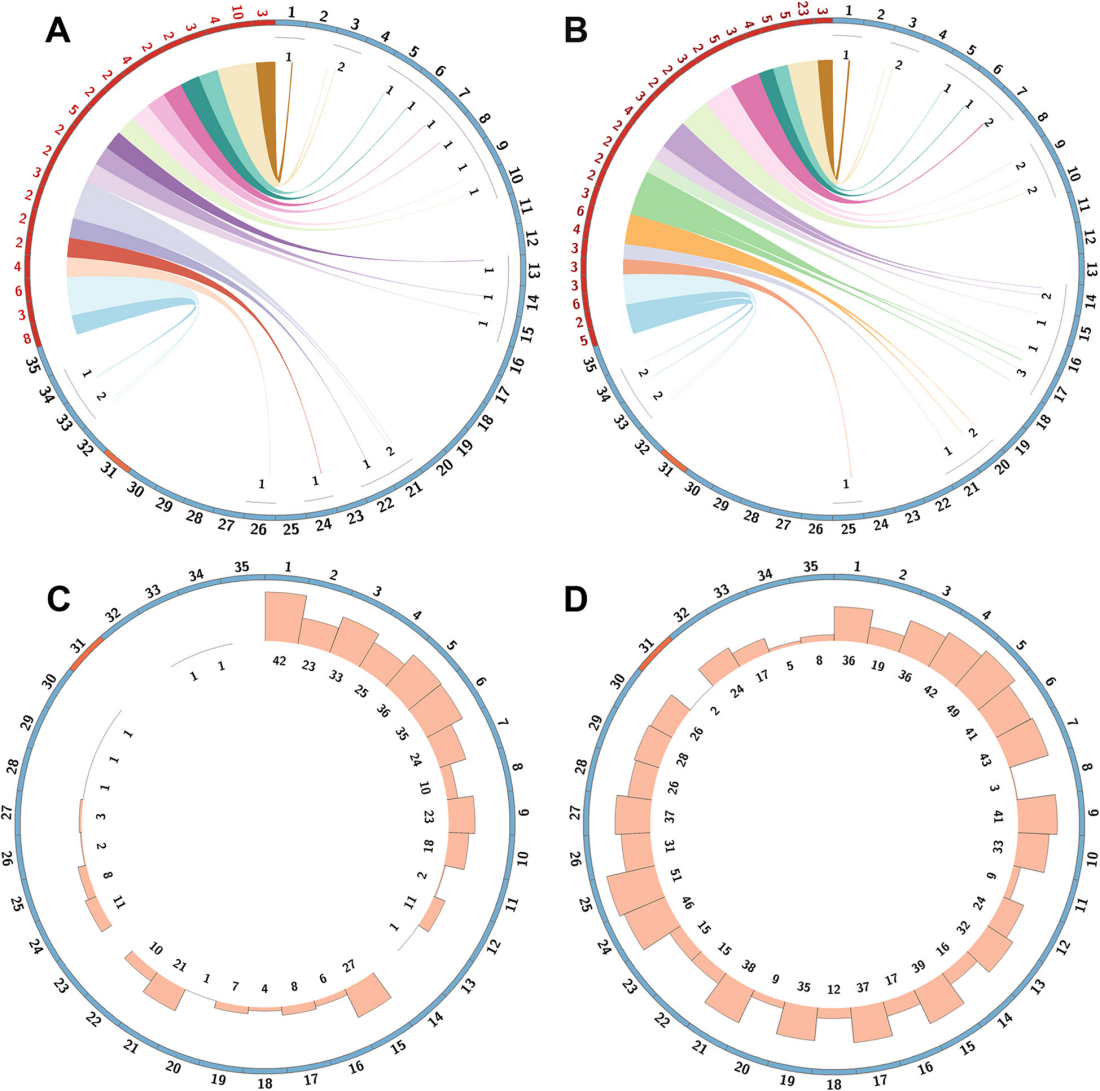
Gene copy number variations in *L. (V.) peruviana.* Mapping of expanded genes in both *L. (V.) peruviana* isolates. **a**, **b** Tandem duplicated gene arrays in PAB-4377 and LEM-1537, respectively. Outer circle shows gene arrays as red with numbers indicating the calculated array expansion. Disomic chromosomes are shown in blue and supernumerary chromosomes in orange with outer numbers describing each chromosome. Colored lines map the location of duplicated arrays in their respective chromosomes. **c**, **d** dispersed duplicated genes in PAB-4377 and LEM-1537, respectively. Histogram and numbers represents the total number of gene expansions in each chromosome

Expanded tandem gene arrays and dispersed genes appear to be a major source of inter and intra-species variation in *Leishmania* [12]. The tandem duplicated gene arrays analysis showed a total of 20 and 26 expanded arrays in PAB-4377 (Fig. 4a) and LEM1537 (Fig. 4b), respectively, relative to the *L. (V.) braziliensis* reference genome (Additional file 1: Table S5).

In both samples, 14 tandem arrays were shared showing that gene array expansions may vary across strains from the same species (Additional file 1: Table S5). The most expanded gene arrays in both isolates belonged to a group of TATE DNA transposons (OG5_128620), NADH-dependent reductases (OG5_128620), heat shock protein 83 (OG5_126623) and hypothetical proteins among others (Additional file 1: Table S5).

The same analysis in *L. (V.) braziliensis* M2904 resulted in 18 tandem gene arrays from which only three arrays were shared with *L. (V.) peruviana* (Additional file 1: Table S6). Interestingly, amastin surface protein arrays that are present in *L. (V.) braziliensis* seems to be not expanded in *L. (V.) peruviana*.

Amastins have been shown to be highly expressed in the amastigote life stage and appear to mediate host-parasite interactions allowing infection and survival [23]. While the effect of this variation remains to be confirmed, these differences may be related with different host interactions in both species.

We found 398 and 942 dispersed duplicated genes in PAB-4377 and LEM1537 with 360 expansions in common (Fig. 4c, d, Additional file 1: Table S7 and S8). Most expanded genes include thioredoxins, NADH-dependent fumarate reductases and several hypothetical proteins.

We did not detect an increase in copy number in GP63 genes in *L. (V.) peruviana* as has been previously shown in *L. (V.) braziliensis* [12] reinforcing a previous finding of GP63 copy number differences between these species [24].

The zinc-metalloprotease GP63 stands out as a major virulence factor in *Leishmania* presenting different roles in the vector and mammal host that aim to protect parasites from host immune responses and promote infection [25]. Therefore, deletion of some GP63 genes in *L. (V.) peruviana* could affect parasite-host interactions and influence its distribution and clinical manifestation with lack of mucosal development.

The marked intra-species difference in dispersed duplicated genes shows that extensive variation in gene copy number can occur between isolates belonging to the same species and supports the hypothesis that chromosome and gene CNV act as a mechanism of rapid parasite adaptation [12, 26].

## Conclusions

Extensive chromosomal and gene copy number variations have been described in *Leishmania* and were proposed as a mechanism of rapid parasite adaptation to different environments and pressures in the host. Our study shows that there are major differences regarding gene copy number variations and aneuploidy even between closely related *Leishmania* species.

Although highly similar to *L. (V.) braziliensis*, *L. (V.) peruviana* presents a different set of expanded gene arrays that can result in different expression profiles between both species. Moreover, high SNP and indel counts as well as extensive variation in chromosome and gene copy numbers between *L. (V.) peruviana* and *L. (V.) braziliensis* support maintaining the classification of both organisms as closely related but distinct species.

Further analysis including a greater number of *L. (V.) peruviana* and *L. (V.) braziliensis* isolates and the use of transcriptomic data are needed to assess if these differences are conserved across isolates of *L. (V.) peruviana* and reveal how tandem gene arrays and CNV affect genome expression.

## Methods

### Parasite isolates and sequencing

*L. (V.) peruviana* isolate PAB-4377 was kindly provided by the U.S. Naval Medical Research Unit No. 6 (NAMRU-6) and the LEM1537 (MHOM/PE/84/LC39) isolate was obtained from the Montepellier reference center.

PAB-4377 was confirmed as *L. (V.) peruviana* by Multilocus Enzyme Electrophoresis (MLEE) and sequencing of the Manose Phosphate Isomerase and 6-phosphogluconate dehydrogenase genes. LEM1537 is a *L. (V.) peruviana* reference strain (MHOM/PE/84/LC39) and has been widely characterized by MLEE.

Libraries consisting of 350 bp fragments were obtained and 100 bp paired end reads were generated at the Genome Technology Access Center (GTAC) at Washington University in St. Louis by Illumina HiSeq 2000. The version 6 of the *L. (V.) braziliensis* M2904 genome was obtained from the Tritryp database (http://tritrypdb.org/) to serve as a reference for comparative analysis.

### Genome assembly and annotation

*L. (V.) peruviana* reads were filtered by quality using Trimmomatic [27] with a minimum base quality cutoff of 30, leading and trailing base qualities of 28, five bases sliding window with minimum per base average quality of 20 and a minimum read length of 70 bp.

A combined *De novo* and reference based assembly approach (Baptista et al., in preparation) was used to generate a draft assembly for each sample. Briefly, *De Novo* assemblies were generated using the Velvet optimizer perl script under Velvet version 1.2.10 [28]. Draft assemblies were extended by iterative mapping using IMAGE [29] and corrected using iCORN2 [30].

For reference-based assembly, reads from each sample were mapped against the *L. (V.) braziliensis* M2904 genome using Bowtie2 [31]. Redundant reads were removed and a reference-based sequence was generated using SAMtools Mpileup and vcfutils [32] using base quality scores greater or equal than 40, mapping quality scores greater or equal than 25, coverage greater or equal than 10 reads and less than twice the median genome coverage.

*De Novo* and referenced based sequences of each sample were combined using the ZORRO hybrid assembler as previously described [33]. The final hybrid assemblies were furthered extended and corrected with IMAGE and iCORN and contigs were scaffolded with SSPACE [34]. Scaffolds were aligned and orientated into pseudochromosomes with ABACAS [35] using the *L. (V.) braziliensis* M2904 genome as a reference sequence.

MUMmer3 [15] was used to calculate similarity between the assembled *L. (V.) peruviana* genomes and the reference *L. (V.) braziliensis.* Briefly, identity scores and number of bases from best local alignments among assembled and reference genomes were retrieved and normalized with the total number of bases in the draft genome in order to compute a global identity score.

Read and assembly files are available through the European Nucleotide Archive under the project number PRJEB7263.

### SNP and pseudogene analysis

To detect SNPs between *L. (V.) peruviana* and *L. (V.) braziliensis* and determine their potential effects on coding sequences, *L. (V.) peruviana* reads were mapped onto the *L. (V.) braziliensis* M2904 reference genome using Bowtie2 and analyzed using the recommended parameters of GATK [36]. Briefly, mapped bam files were filtered for redundant reads and local realignment was performed around indels in order to remove potential mapping artifacts. SNPs were called using the haplotype caller module and raw variants were filtered using GATK’s variant quality score recalibration selecting sites with a minimum raw coverage of 10, Root Mean Square mapping quality lower than 40, quality by depth greater than 2 and haplotype score greater than 13. The same method was employed to call variants between both *L. (V.) peruviana* isolates.

To analyze the effects of SNPs in coding regions of the *L. (V.) peruviana* genome, we filtered variant calls of PAB-4377 and LEM1537 selecting only SNPs shared by both isolates to limit the potential impact of within-species SNP variability and minimize incorrect SNP calling. The combined variant called was used as input for SnpEff [37] to annotate and predict the effects of variants of genes.

To find potential targets sequences to accurately discriminate *L. (V.) peruviana* from *L. (V.) braziliensis* we employed a custom Perl script to screen the genes with variant calls. These genes were analyzed using a sliding window of 1000 nucleotides to report the region with the highest SNP density and the number of SNP that it presented. Genes with significant SNP calls were detected using the ROUT test under Graph Pad Prism V5 [38]

We downloaded *L. (V.) braziliensis* pseudogenes from the Tritryp database and compared them against *L. (V.) peruviana* to detect potential pseudogenes that remained functional in *L. (V.) peruviana*. Briefly, *L. (V.) peruviana* amino acid fasta sequences were generated using SAMtools Mpileup and translated into amino acids for sequence alignment against *L. (V.) braziliensis* pseudogenes in ClustalΩ [39].

### Allele frequency distribution

Allele frequencies for PAB-4377 and LEM1537 assemblies were obtained from filtered SAMtools Mpileup results as described elsewhere [12]. Briefly, the proportion of reads mapping to each heterozygous site under the total mapped reads for the site was estimated. Allele frequencies were categorized from 0.1 to 1.0 and normalized by the sum of all allele frequencies for the chromosome. Allele frequencies distributions were plotted in R and plots from chromosomes sharing the same pattern were combined.

### Chromosome and tandem gene array analysis

To analyze chromosome copy number, we combined three different approaches based on the assumption that the overall chromosome organization is similar between *L. (V.) braziliensis* and *L. (V.) peruviana*. First, OrthoMCL was used to select single copy genes from the proteomes of *L. (V.) braziliensis*, *L. (L.) mexicana* and *L. (L.) major*, *L. (L.) infantum, L. (L.) donovani* and *L. (Sauroleishmania) tarentolae* (Additional file 1: Table S9).

This group of single copy genes was used to normalize read mapping counts for each position along the chromosome in order to calculate haploid copy number. Second, the number of reads mapping to the whole chromosome was counted and normalized by the median number of mapped reads to the whole genome. Third, we normalized FPKM (Fragments Per Kilobase per Million fragments mapped reads) values for each chromosome by the median FPKM of the whole genome. We plotted the mean and standard deviations from the three approaches using Graph Pad Prism V5.

We normalized haploid copy numbers with the average chromosome ploidy calculated from the allele frequency analysis to estimate chromosome ploidy. Plots for each chromosome were generated in R using a sliding window of 10 kilo bases.

Gene Ontology codes that were significantly overrepresented in the genes of supernumerary chromosomes were detected using the hypergeometric distribution analysis in BiNGO [40] with Benjamini and Hochberg false discovery rate correction.

We defined tandem gene arrays as groups of genes that are located contiguously in a chromosome and that share a homology relationship. Dispersed gene duplications are defined as genes that are duplicated and do not belong to any tandem array.

Dispersed and tandem gene duplications were identified using a combination of Bowtie2 and Cufflinks2 [41]. Briefly, mapped reads against *L. (V.) braziliensis* M2904 and a coding sequence (CDS) GFF file were used as input for Cufflinks2 to determine FPKM for each CDS and chromosome. Haploid copy number for each CDS was estimated by a proportion of their respective FPKM and the median FPKM of all CDS in the respective chromosome. We employed OrthoMCL [42] to identify homology relationships in mapped CDS and the mean haploid copy number was estimated for each array as reported by Rogers [12]. Gene duplications were defined as those greater than a cutoff of 1.85 for the haploid number computed by our analysis [12].

We employed this same approach to detect expanded gene arrays in the *L. (V.) braziliensis* genome using reads from the M2904 reference strain.

## Additional files

Additional file 1:
**Supplementary tables.** (XLSX 1072 kb) Additional file 2:
**Top five high SNP density genes.** (TIFF 448 kb) Additional file 3:
**Normalized read depth for PAB-4377 chromosomes.** (TIFF 2222 kb) Additional file 4:
**Normalized read depth for LEM-1537 chromosomes.** (TIFF 2449 kb) Additional file 5:
**Normalized allele frequency distributions for PAB-4377 chromosomes.** (TIFF 443 kb) Additional file 6:
**Normalized allele frequency distributions for LEM-1537 chromosomes.** (TIFF 441 kb)
