# Supplementary figures and images for: Comparative genomic analysis of Leishmania (Viannia) peruviana and Leishmania (Viannia) braziliensis
Source: BMC Genomics. 2015 Sep 18;16(1):715. doi: 10.1186/s12864-015-1928-z (PMC4575464; doi:10.1186/s12864-015-1928-z)

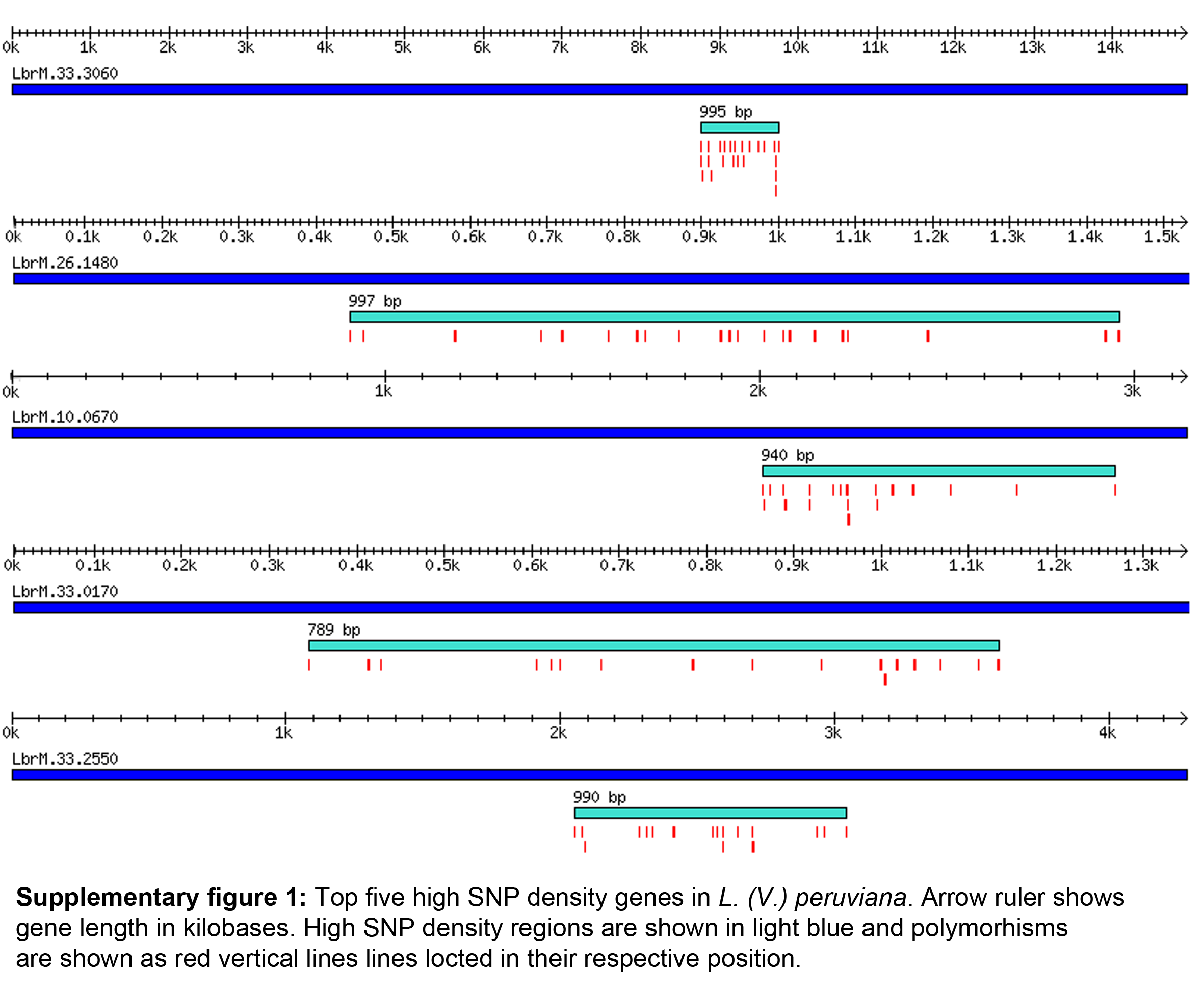

Supplement: Additional file 2: — Top five high SNP density genes. (TIFF 448 kb) [file 12864_2015_1928_MOESM2_ESM.tiff]

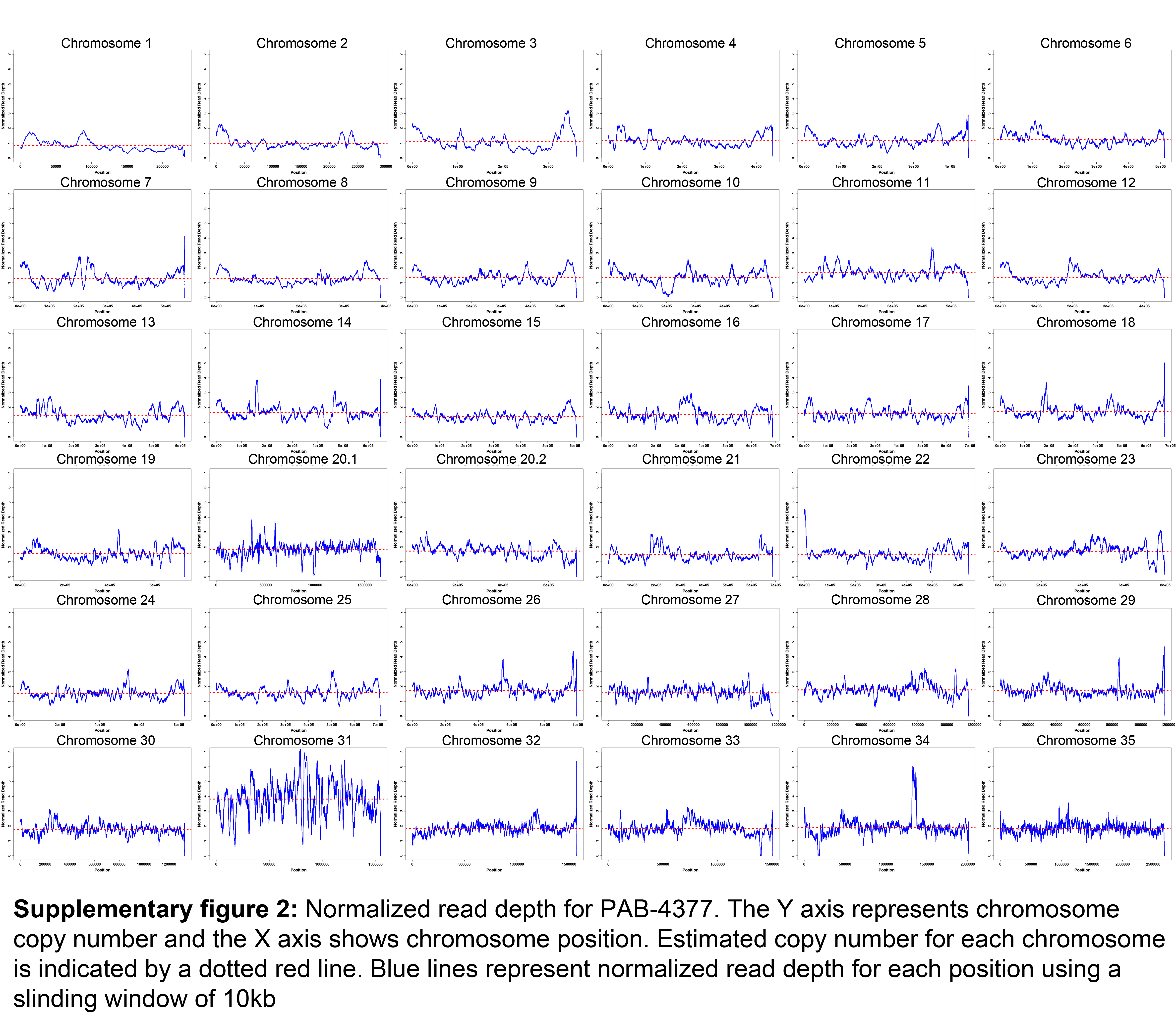

Supplement: Additional file 3: — Normalized read depth for PAB-4377 chromosomes. (TIFF 2222 kb) [file 12864_2015_1928_MOESM3_ESM.tiff]

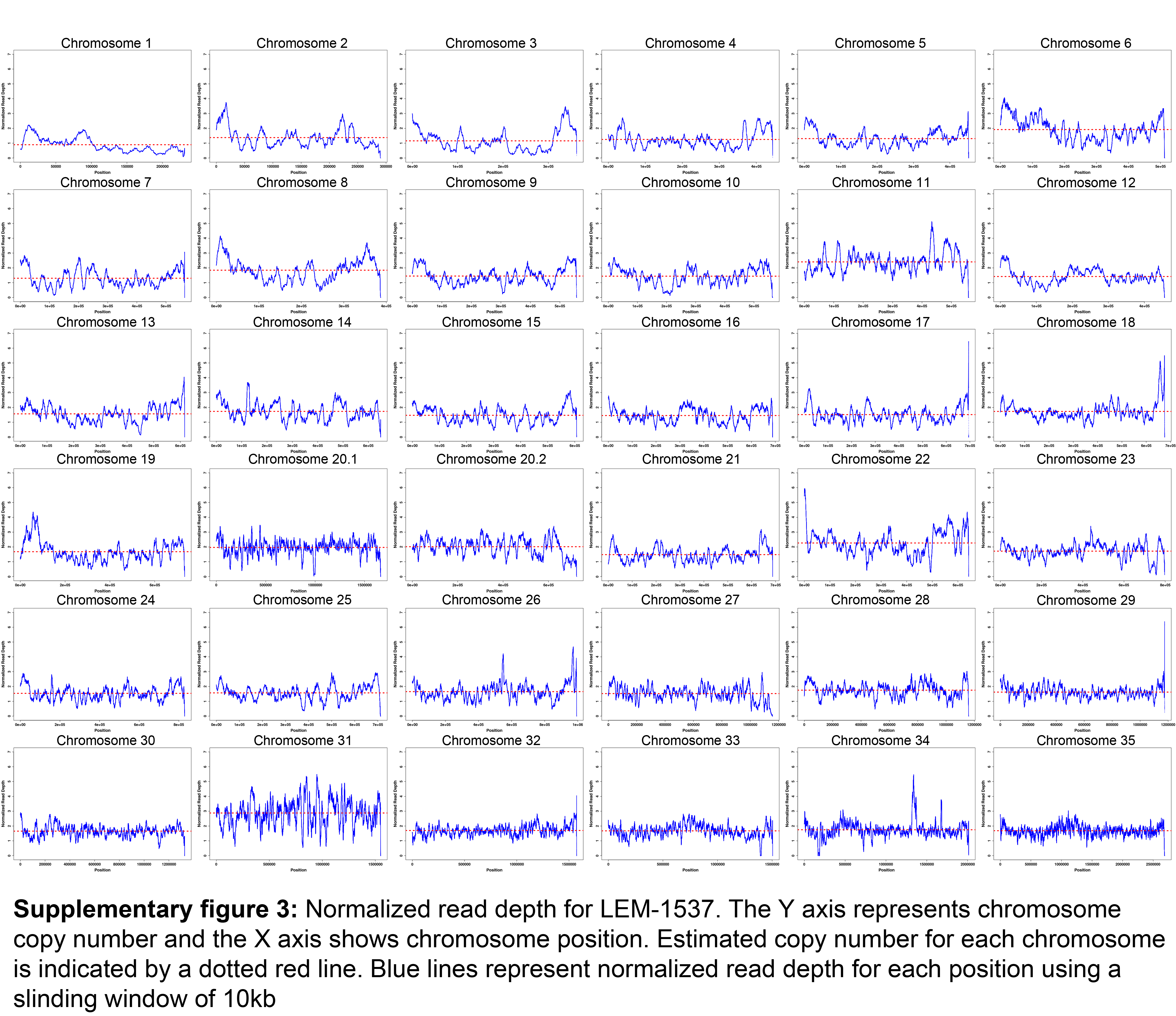

Supplement: Additional file 4: — Normalized read depth for LEM-1537 chromosomes. (TIFF 2449 kb) [file 12864_2015_1928_MOESM4_ESM.tiff]

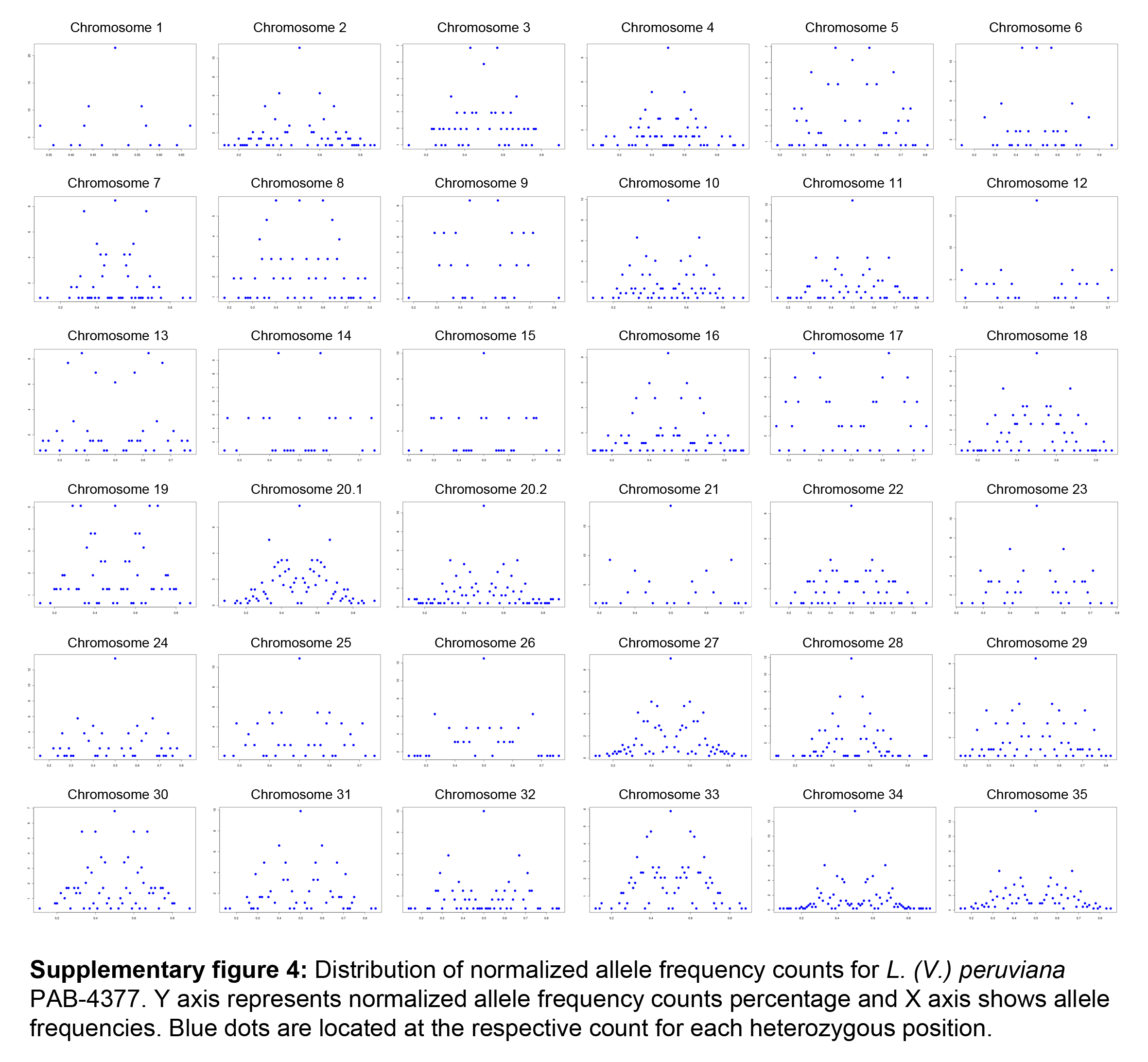

Supplement: Additional file 5: — Normalized allele frequency distributions for PAB-4377 chromosomes. (TIFF 443 kb) [file 12864_2015_1928_MOESM5_ESM.tiff]

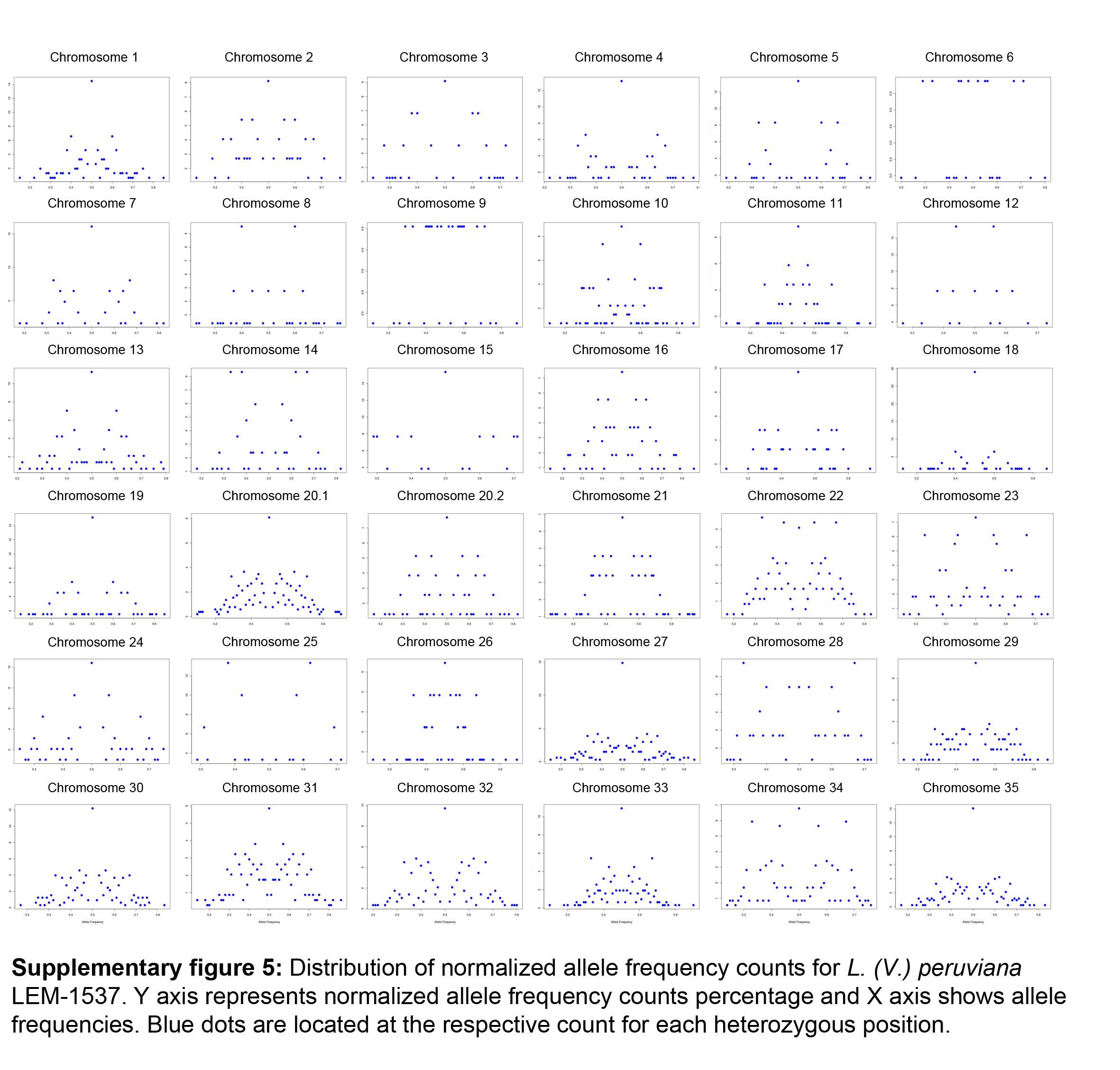

Supplement: Additional file 6: — Normalized allele frequency distributions for LEM-1537 chromosomes. (TIFF 441 kb) [file 12864_2015_1928_MOESM6_ESM.tiff]
